# Predeliberation activity in prefrontal cortex and striatum and the prediction of subsequent value judgment

**DOI:** 10.3389/fnins.2013.00225

**Published:** 2013-11-26

**Authors:** Uri Maoz, Ueli Rutishauser, Soyoun Kim, Xinying Cai, Daeyeol Lee, Christof Koch

**Affiliations:** ^1^Division of Biology, California Institute of TechnologyPasadena, CA, USA; ^2^Department of Neurosurgery and Neurology, Cedars Sinai Medical CenterLos Angeles, CA, USA; ^3^Department of Neurobiology, Yale University School of MedicineNew Haven, CT, USA; ^4^Department of Anatomy & Neurobiology, Washington UniversitySt. Louis, MO, USA; ^5^Allen Institute for Brain ScienceSeattle, WA, USA

**Keywords:** pre-deliberation decision bias, value-based decision-making, decision circuit-modeling, free-choice decision making, dorsolateral prefrontal cortex, ventral striatum, caudate nucleus, monkey single-neuron recording

## Abstract

Rational, value-based decision-making mandates selecting the option with highest subjective expected value after appropriate deliberation. We examined activity in the dorsolateral prefrontal cortex (DLPFC) and striatum of monkeys deciding between smaller, immediate rewards and larger, delayed ones. We previously found neurons that modulated their activity in this task according to the animal's choice, while it deliberated (choice neurons). Here we found neurons whose spiking activities were predictive of the spatial location of the selected target (spatial-bias neurons) or the size of the chosen reward (reward-bias neurons) before the onset of the cue presenting the decision-alternatives, and thus before rational deliberation could begin. Their predictive power increased as the values the animals associated with the two decision alternatives became more similar. The ventral striatum (VS) preferentially contained spatial-bias neurons; the caudate nucleus (CD) preferentially contained choice neurons. In contrast, the DLPFC contained significant numbers of all three neuron types, but choice neurons were not preferentially also bias neurons of either kind there, nor were spatial-bias neurons preferentially also choice neurons, and vice versa. We suggest a simple winner-take-all (WTA) circuit model to account for the dissociation of choice and bias neurons. The model reproduced our results and made additional predictions that were borne out empirically. Our data are compatible with the hypothesis that the DLPFC and striatum harbor dissociated neural populations that represent choices and predeliberation biases that are combined after cue onset; the bias neurons have a weaker effect on the ultimate decision than the choice neurons, so their influence is progressively apparent for trials where the values associated with the decision alternatives are increasingly similar.

## Introduction

There is a long-standing debate on the proper interpretations of brain signals that predict upcoming free decisions before subjects report having decided. In such free decisions the choice is up to the subject and there is no right or wrong answer (Libet et al., 1983; Libet, 1985). Recent results suggest that motor intentions can be decoded in these cases from neural activity up to about a second before action onset using EEG (Haggard and Eimer, 1999; Bai et al., 2011) or single-neuron activity in humans (Fried et al., 2011) and up to several seconds before onset, at about 60% accuracy, using functional magnetic-resonance imaging (fMRI; Soon et al., 2008; Bode et al., 2011). It was also demonstrated that more-abstract decisions (such as adding or subtracting two small numbers) could be decoded before the decision alternatives became available (Haynes et al., 2007; Soon et al., 2013). Interestingly, models of these experimental results focus on the dorsolateral prefrontal cortex (DLPFC) as the locus of intentional action selection (Pockett, 2006; Pacherie and Haggard, 2011).

However, the choices in the above experiments were generally between randomly raising the left or right hand, with no consequences and for no reason or purpose (although early, predictive local-field potentials may also exist for deliberate decision making that relies on rational deliberation; Maoz et al., 2012). So, importantly, it is not clear to what extent these early predictive signals are part of the neural process involved in deliberate decisions. An alternative hypothesis is that in the absence of reasons to prefer one option over the other, the decision network may also rely on activity that occurs before the decision options are known and rational deliberation can begin. Such slow background fluctuations, for example, could be used to break the symmetry and bias the decision toward one of the equally valued alternative (Haynes, 2011). In contrast, for divergently valued alternatives, the predeliberation fluctuations that occur before rational deliberation can begin are generally not strong enough to influence the outcome, so they would have little predictive power over the decisions. An empirical prediction of this predeliberation-biases hypothesis is, therefore, that outcomes of decisions between similarly valued options could be, to some extent, predicted from brain activity occurring before the subject knows what the decision alternatives will be.

Neuronal activity before the presentation of the decision alternatives that is predictive of the eventual choice has been shown before, including in prefrontal cortex and the caudate nucleus (CD). But this was in situations where the decision alternatives associated with maximal reward were stable across multiple trials, and the optimal choice could thus be computed before the decision alternatives were revealed (Coe et al., 2002; Lauwereyns et al., 2002; Ding and Hikosaka, 2006). Perceptual judgments were also shown to partially depend on ongoing neural activity prior to the onset of the sensory stimuli (Sadaghiani et al., 2010), including in CD—but were not found in the DLPFC (Kim and Shadlen, 1999; Shadlen and Newsome, 2001; Williams et al., 2003; Ding and Gold, 2010). Moreover, an integrate-and-fire attractor model of decisions based on random spontaneous firing suggested that decisions can be predicted before cue onset from ongoing noise (Rolls and Deco, 2011). However, little is known about the neural correlates of biases during value-based decision-making, where computation of the optimal decision cannot begin before the decision alternatives are presented. In this type of decisions subjects' choices are freely based on the subjective values they associate with the decision alternatives (Rangel et al., 2008), and it is difficult to define correct and incorrect responses.

A common example of conflict among value-based decision options is intertemporal choice between a small, immediate reward and a larger, delayed one (Frederick et al., 2002). Previous studies found that the activity of individual neurons in the DLPFC, parietal cortex, and basal ganglia (BG) encode the subjective value of a delayed reward, namely, the magnitude of reward discounted by its delay (Kim et al., 2008; Louie and Glimcher, 2010; Cai et al., 2011). But how such temporally discounted values are used for decision making in the brain is not well understood. The prefrontal cortex, including DLPFC, has been implicated in multiple aspects of decision making in both humans and non-human primates (Rangel et al., 2008; Wallis and Kennerley, 2010), such as coding subjective values for alternative options or coding the option chosen by the animal (Barraclough et al., 2004; Padoa-Schioppa and Assad, 2006; Kennerley et al., 2009). Previous neuroimaging, lesion, and stimulation studies also highlighted the role of BG and cortico-BG communication in regulating decisions involving temporal delays (Kable and Glimcher, 2007, 2010; Luhmann et al., 2008; Pine et al., 2009; Cavanagh et al., 2011).

Following the above, we therefore focused here on an intertemporal choice in value-based decision-making to empirically test a prediction of the predeliberation-biases hypothesis above. Namely, we wanted to investigate the extent to which neural information that was represented in the DLPFC, ventral striatum (VS) and CD before cue onset—hereafter *bias activity*—influenced later decisions. We also wanted to know whether there was variability in bias-activity representation across brain regions. We were further interested in how bias activity differs, if at all, from value-related information that appeared after cue onset during deliberation—hereafter *choice activity*—and how the two are integrated. Our working hypothesis was that the bias activity was dissociated from the choice activity, and had a weaker influence on the ultimate decision than that choice activity, so that its effect was mainly apparent when the values of the decision alternatives were similar.

## Materials and methods

### Animal preparation

All data analyzed in this study were from three male rhesus monkeys (D and J for DLPFC, H and again J for BG). Behavioral and neurophysiological data were collected as previously described (Kim et al., 2008; Cai et al., 2011). All procedures used in the present study were approved by the Institutional Animal Care and Use Committee at Yale University and The University of Rochester Committee on Animal Research, and conformed to the *PHS Policy on Humane Care and Use of Laboratory Animals* and the *Guide for the Care and Use of Laboratory Animals*.

### Behavioral task

**Figure 1:**
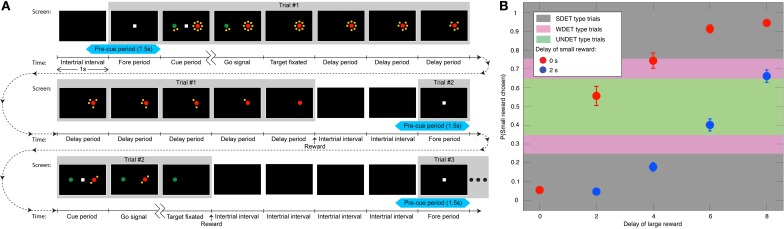
**Task and behavior. (A)** Three animals (two in the DLPFC experiment and one of the two and another in the basal-ganglia experiment) indicated their preference between a smaller and more immediate juice reward (designated by the green target) and a larger, but generally delayed one (red target). The number of yellow dots surrounding the targets—the clock—indicated the delay in seconds associated with each target. Portrayed are the full timelines and screenshots of two trials and the beginning of a third trial. Each trial is made up of a 1 s fore period, followed by a 1 s cue period, and afterwards a go signal leading to target fixation. Then, after the delay associated with the chosen target is counted down, the reward is delivered. And, after an inter-trial interval of 2 s, the next trial starts. If the animal chooses the target with the shorter delay, the difference between the longer and shorter delay is added to the inter-trial interval, so as not to motivate the animal to select the target with the shorter delay to bring about the next trial more quickly. The first trial depicts a choice between a 2 s-delay small reward and 8 s-delay large one, where the animal selects the large reward. In the second trial, the animal opts for an immediate small reward over a 2 s-delayed large reward. The interval between two ticks on the time scale is 1 s. The 1.5 s pre-cue period is denoted in light blue. **(B)** The mean probability (±SEM) of choosing the small reward vs. the 9 possible combinations of delays (*N* = 62 sessions) from both animals in the DLPFC, including the UNDET, WDET, and SDET probability ranges.

The animals performed an intertemporal choice and a control task. During each trial of the intertemporal choice task, the animal fixated a small white square that appeared at the center of the screen during a 1 s fore period (Figure 1A). Two peripheral targets were next presented along the horizontal meridian during the 1 s cue period. The central square was then extinguished—the go signal—and the animal was required to shift its gaze toward one of the two targets. Choosing the red target resulted in a large reward (0.4 ml of apple juice), while the green target delivered a smaller reward (0.26 ml). Target positions were randomized and counterbalanced across trials. Each target appeared either by itself or surrounded by a clock consisting of 2, 4, 6, or 8 yellow dots. This corresponded to no delay or to 2, 4, 6, or 8 s delay before reward delivery, respectively. Once the animal fixated its chosen target, the other target disappeared, and the clock surrounding the chosen target began counting down the dots at a rate of 1/s (Figure 1A). The animal was rewarded when the last yellow dot disappeared. The screen then went blank for a 2 s inter-trial interval. When the animal chose the target with the shorter delay, the inter-trial interval was increased by the difference between the longer and shorter delay, so that the onset of the next trial was not influenced by the animal's choice (Figure 1A). The delay associated with the small-reward target was 0 or 2 s long, and that associated with the large-reward was 0, 2, 4, 6, or 8 s. For the striatum data all 10 possible delay pairs were used, while for the DLPFC the combination of 2 s delay for the small reward and 0 s delay for the large reward was not used. Each combination was presented twice (four times) in a block of DLPFC (striatum) intertemporal choice trials with the target positions counterbalanced; 18 (40) trials per block in experiments in the DLPFC (striatum).

The control task was identical to the intertemporal choice task, except for two changes. First, the central fixation square was either green or red, which informed the animal of the target it was required to select. Incorrect trials were not rewarded. Second, the reward amount was fixed (at 0.13 ml) and awarded with no delay. Blocks of intertemporal choice and control trials were presented alternately. The experiment was set up so that each session would consist of the same number of trials in the intertemporal choice and control tasks. Of the 164 DLPFC neurons, 135 were tested in 10 blocks for each condition (360 trials) and the rest were tested for at least six blocks for each condition (216 trials). Of the 183 striatal neurons (93 CD, 90 VS), 181 were tested over at least three blocks (120 trials) and on average more than four blocks were tested per neuron (167.4 ± 3.7 and 162.4 ± 4.1 trials for CD and VS, respectively).

When the animal broke fixation before the go signal, the trial was restarted with the same rewards and delays. If fixation was broken after cue onset (in 4855 of overall 36738, 13%, and in 369 trials of overall 34052 trials, 1%, for DLPFC and striatal recordings, respectively), the animal could have gained information about the decision option it was about to face in the upcoming restarted trial. Trials that immediately followed those where fixation breaking occurred after cue onset were therefore not included in the analysis. This, together with the randomization of the small and large reward positions and their associated delays for the intertemporal choice and control blocks, meant that during the pre-cue period analyzed in the present study, the animal would know neither the positions nor the delay of the targets about to appear. Therefore, in our task, cue onset is also when rational deliberation can begin. Our data is taken from Experiment I of Kim et al. (2008) and from Cai et al. (2011), where more details can be found.

### Analysis of behavioral data—partitioning the trials into four types

Following our working hypothesis, we expected the bias activity to increasingly influence the animals' decisions when the subjective values that the animals associated with the decision alternatives became more similar—i.e., when the temporally discounted reward magnitudes associated with the two targets were comparable for the animal. In other words, the less the decision was determined by the cue information, the more we expected it to be predictable from the bias activity. So we divided the experimental trials into four types according to the apparent similarity of the values of the decision alternatives. The frequency with which the animals chooses a particular target over its alternative within every pair of reward/delay combinations is a well-established estimate of the subjective value of that option (Sugrue et al., 2004). We therefore used it here too. More specifically, the animals increasingly discounted the value of the reward as a function of the reward's delay (Figure 1B) (Kim et al., 2008; Hwang et al., 2009). For each recording session, there were 9 or 10 possible short/long delay pairs (see above), each associated with the ratio of trials in which the animal chose the small reward to the overall number of trials (Figures 1B, Figure S1). The four trial types were: The *undetermined* trials [where the probability of selecting the small reward was in the range (0.35, 0.65)]—denoted *UNDET*—that had the most similar discounted values for the two targets, making the decision largely undetermined by the visual cues. The *weakly determined* trials [choice ratio range (0.25,0.35) or (0.65,0.75)]—*WDET*—that had the discounted values for the two targets farther apart. The *strongly determined* trials [ratio in (0,0.25) or (0.75,1)]—denoted *SDET*—that had the most divergent discounted values. Lastly, *no choice* trials—*NC*—included all trials in the control experiment, where the animal was instructed which target to fixate.

### Analysis of neural data

Overall, 164 DLPFC neurons were recorded (77 from animal D, 87 from J), 90 VS neurons (33 from H, 57 from J), and 93 CD neurons (32 from H, 61 from J). Great care was taken to insure that all spikes originated from single neurons. To determine bias neurons we looked at spiking activity 1.5 s before cue onset in all analyses—henceforth the *pre-cue period*. This time frame was comprised of the last 0.5 s of the inter-trial interval and the 1 s fore period (Figure 1A). We included only the last half-second of the inter-trial interval because we did not want activity from the previous trial, which might linger in the beginning of the inter-trial interval, to confound our results.

In addition, only neurons that satisfied three criteria were included in this study. First, they were recorded during sessions containing at least one UNDET-type reward-delay combination (which on average ± SEM consisted of 32 ± 3, 23 ± 3, and 30 ± 6 trials for the DLPFC spatial-bias, DLPFC reward-bias, and VS spatial-bias, respectively). Second, the mean firing rate of the neurons 1.5 s before cue onset was at least 0.5 spikes/s. Third, the animal did not display a very strong behavioral spatial preference toward the left or the right—i.e., it had to choose the left and right target at least 25% of the trials. Importantly, any deviations from an even left/right split are specifically taken into account by our method for identifying bias neurons and by the proportion explained measure (see below). When analyzing the DLPFC data for spatial biases, 105 of the 164 neurons (64%) met all three criteria and were subjected to further analyses (44 from animal D). In the analysis of reward biases, the third criterion was not imposed because it is dissociated from a choice based on reward size. Hence, for the reward-size analysis, 118 of the 164 neurons (72%) were subjected to further analysis (53 from animal D). For VS, 52 and 56 of the 90 neurons (58 and 62%) were included in the spatial-bias and reward-bias analyses (38 and 40 from J), respectively. For CD, 54 and 60 of the 93 neurons (58 and 65%) were included in the spatial-bias analysis and reward-bias analyses (43 and 44 from J), respectively.

The 105 DLPFC neurons used in the spatial-bias analysis were recorded over 62 sessions. Given our neuron inclusion criteria, there was at least one UNDET-type reward/delay combination in each session, but only 30 of the 62 sessions (48%) also contained WDET-type trials. The 118 DLPFC neurons used in the reward-bias analysis were recorded over 69 sessions. WDET-type trials appeared in 35 of those 69 sessions (51%). The 52 VS neurons were recorded over 51 sessions, 34 of which (65%) also contained WDET-type trials. The VS did not contain more reward-bias neurons than expected by chance and the CD did not contain more spatial- or reward-bias neurons than expected by chance (Table S1). So those breakdowns are not included here.

**Figure 2:**
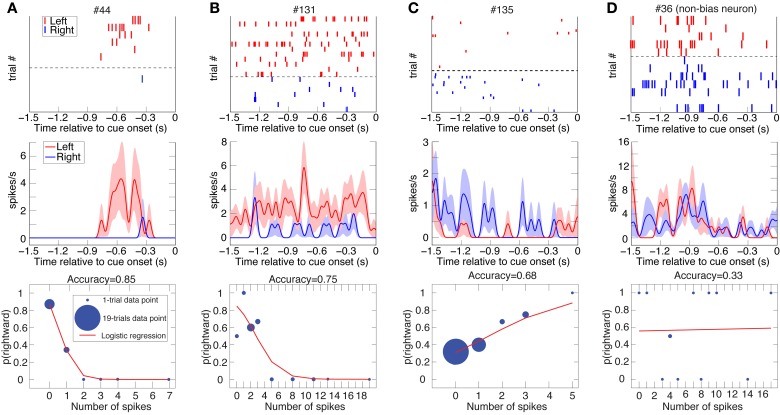
**Three examples of DLPFC spatial-bias neurons and one non-bias neuron.** Raster plots (top; ranked by increasing trial number for trials with subsequent leftward and rightward eye movements separately), spike-density function plots (mean ± SEM; middle), and logistic-regression best-fits (bottom) to the data of 4 DLPFC neurons (Panels **A** and **D** from Animal D, **B** and **C** from J). Cue onset is at 0 s, so depicted is spiking activity from the last half-second of the inter-trial interval as well as from the 1 s fore period. The data is taken from all the undetermined (UNDET) trials of each neuron, because bias neurons were selected using only UNDET trials. Panels **(A–C)** depict spatial bias neurons, while panel **(D)** is a non-bias neuron. The number of trials associated with each data point in the logistic regression is designated by the diameter of the symbol. (See Figure S2A for ROC and choice-probability analyses of these neurons.).

**Figure 3:**
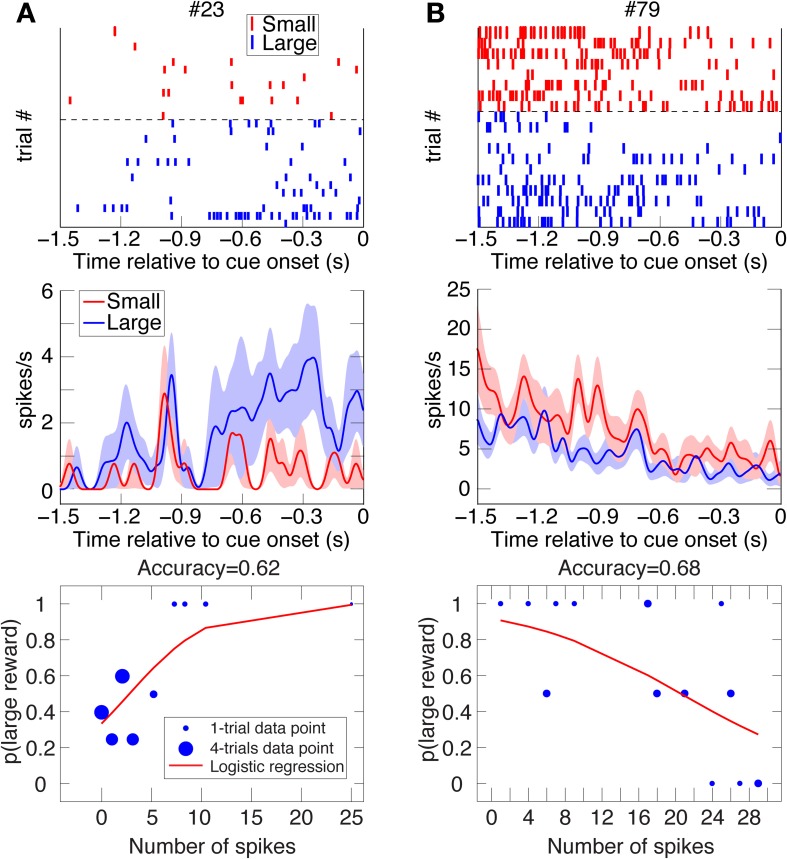
**Two examples of DLPFC reward-bias neurons.** Raster plots, spike-density function plots (mean ± SEM), and logistic-regression best-fits to the data of 2 DLPFC reward-bias neurons from all their UNDET trials (Panels **A** and **B** from Animal D and J, respectively). Details are given in Figure 2. (See Figure S2B for ROC and choice-probability analyses of these neurons.).

**Figure 4:**
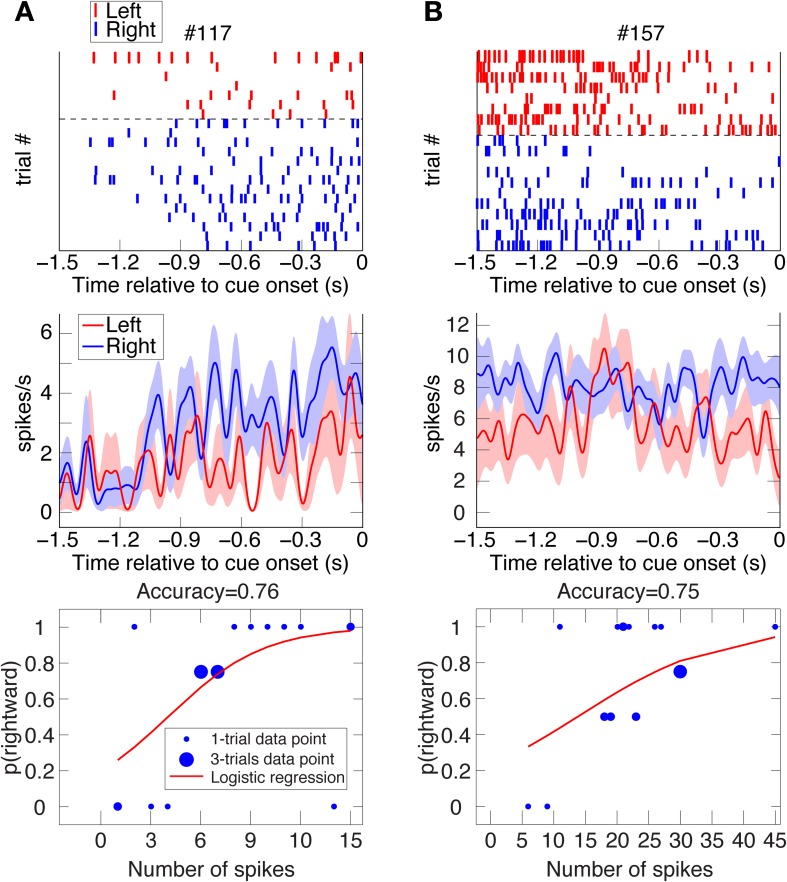
**Two examples of VS spatial-bias neurons.** Raster plots, spike-density function plots (mean ± SEM), and logistic-regression best-fits to the data of 2 VS reward-bias neurons from all their UNDET trials (Panels **A** and **B** from Animal H and J, respectively). Details are given in Figure 2. (See Figure S2C for ROC and choice-probability analyses of these neurons.).

For each of the participating neurons, we counted the number of spikes in the 1.5 s before cue onset—during the last 0.5 s of the inter-trial interval and the entire 1 s fore period (Figure 1A)—for every participating trial. Spike bursts—action potentials fired less than 10 ms apart—were consolidated into a single spike (Bair et al., 1994) (omitting this step did not affect any of our main result). We also computed a spike density function (SDF) for each spike train by convolving it with a 100 ms Gaussian window (Figures 2–4), which was used for plotting purposes only.

#### Computing prediction accuracy, proportion explained, and bias neurons

Counting the number of spikes during the 1.5 s prior to cue onset in each trial, we calculated the ratio of trials culminating in rightward saccades or high-reward choices to overall trials for each unique number of spikes (Figures 2–4). Analyzing each neuron separately, we then used binomial logistic-regression in a leave-one-out cross-validation procedure—training the model on all but one trial and testing it on the remaining, statistically independent trial—to predict rightwards saccades or large-reward choice for the remaining test-set trial. We labeled left (low-reward) as 0 and right (high-reward) as 1, and predicted a leftward (low-reward) choice for a trial if the predicted probability from the logistic regression was <0.5 and rightward (high-reward) otherwise (a log-likelihood-ratios analysis of the predicted probabilities suggested that 0.5 was the optimal threshold to use for our data). The prediction accuracy for each neuron was defined as the ratio of correctly predicted trials to overall trials.

For the spatial-bias analysis, the numbers of left and right choices for each neuron were not generally similar (Figures 2–4), while the number of high- and low-reward choices for each neuron tended to be more similar. Therefore, chance-level prediction varied among the neurons according to the behavioral preference of the animal, and we computed it using bootstrap resampling of the original data. For each neuron we randomly shuffled the left and right labels or high- and low-reward labels among the trials and computed the prediction accuracy with the same logistic regression method. We repeated this procedure 10,000 times to create an expected distribution of chance prediction-accuracy (Efron and Tibshirani, 1993). The mean of this chance distribution was designated the chance-level prediction-accuracy for this neuron.

**Figure 5:**
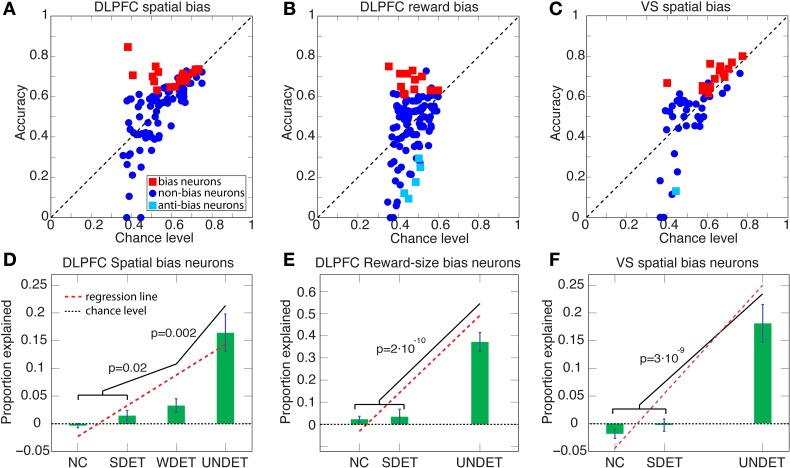
**Accuracy vs. chance level and proportion explained above chance level for DLPFC spatial- and reward-bias neurons and VS spatial-bias neurons. (A–C)** Scatter plots of accuracy vs. chance level are depicted for UNDET trials. Bias neurons and anti-bias neurons (see Figure 6) are designated in red and cyan squares, respectively. **(D)** Average (±SEM) proportion explained above chance level over DLPFC spatial-bias neurons during UNDET, weakly determined (WDET), strongly determined (SDET), and no-choice control (NC) trials. The proportions explained for UNDET (0.16 ± 0.03; ±SEM) and WDET (0.03 ± 0.01) are significantly greater than expected by chance, while those at SDET (0.01 ± 0.01) and NC (−0.005 ± 0.004) are at chance (paired one-tailed *t*-test, *p* = 2 · 10^−5^, *p* = 0.01, *p* = 0.09, and *p* = 0.9, respectively). In addition, the slope of the regression line through the four trial types was significantly positive (*p* < 2 · 10^−8^). **(E)** Average (±SEM) proportion explained above chance level over DLPFC reward-bias neurons during UNDET, SDET NC trials. The proportion explained for UNDET (0.37 ± 0.04) was significantly greater than expected by chance while the SDET (0.03 ± 0.03) an NC (0.02 ± 0.01) ones were not (paired one-tailed *t*-test, *p* = 2 · 10^−6^, *p* = 0.17, and *p* = 0.06, respectively). The slope of the regression line through the data was also significantly positive (*p* < 4 · 10^−7^). WDET trials were not included because there were too few trials—only 5 of 12—for meaningful statistical analysis. **(F)** Average (±SEM) proportion explained above chance level over VS spatial-bias neurons during UNDET, SDET, and NC trials. The proportion explained for UNDET (0.17 ± 0.03) was significantly greater than expected by chance, while the SDET (−0.004 ± 0.01) and NC (−0.02 ± 0.01) values were not (paired one-tailed *t*-test, *p* = 8 · 10^−5^, *p* = 0.65, and *p* = 0.9, respectively). The slope of the regression line through the data was also significantly positive (*p* < 1 · 10^−6^). WDET trials were not included because there were too few trials—only 9 of 14—for meaningful statistical analysis. *P*-values in the figure are for one-tailed *t*-tests. (See Figure 6 for histograms of the percentiles of the prediction accuracies in the chance-level distributions.).

**Figure 6:**
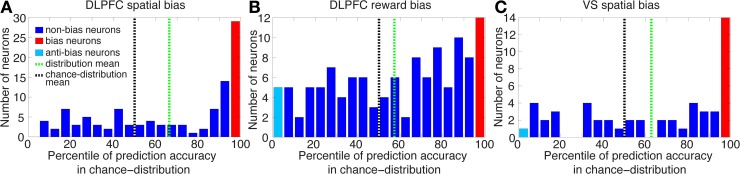
**Histograms of percentiles of prediction accuracies in the distributions of chance level over the entire neuron populations.** For each neuron in the population we calculated its prediction accuracy, and then by repeatedly and randomly shuffling its left/right or large-/small-reward labels also its chance-level distribution. We then computed in which percentile of its chance-level distribution the neuron's prediction accuracy lay. A histogram of these percentiles over all neurons is plotted here. Neurons at the top/bottom 5-percentile are designated bias/anti-bias neurons, respectively. **(A)** The mean percentile of all neurons involved in DLPFC spatial-bias analysis (*n* = 105) is 66% (green dotted line), and is significantly greater than chance-distribution mean (at 50%, black dotted line; Wilcoxon test *p* = 2 · 10^−7^). Also, 69 of the 105 DLPFC neurons are in the top 50-percentile of the chance-distribution mean, which is significantly more than expected by chance (binomial test *p* = 8 · 10^−4^). **(B)** The mean percentile of all neurons involved in DLPFC reward-bias analysis (*n* = 118) is 57% and significantly greater than the chance-distribution mean (Wilcoxon test *p* = 0.01); 70 of 118 neurons are in the top 50-percentile, significantly more than expected by chance (binomial test *p* = 0.026). **(C)** The mean percentile of all neurons involved in VS spatial-bias analysis (*n* = 52) is 63% and significantly greater than chance-distribution mean (Wilcoxon test *p* = 0.004); 33 of 52 neurons are in the top 50-percentile, significantly more than would be expected by chance (binomial test *p* = 0.035).

We computed the proportion of the variance that remained unexplained above chance level and was explained by the activity of the neuron—termed *proportion explained*—for all trial types. More specifically, designating the prediction accuracy by *a*_*i*_ and chance level by *c*_*i*_ for neuron *i*, we calculated (*a*_*i*_ − *c*_*i*_)/(1 − *c*_*i*_), where the denominator, (1 − *c*_*i*_), is the variance left unexplained by the chance level. For example, in a session where the monkey saccaded to the left 65% of the time, a neuron predicting 75% of the trials correctly would have a proportion explained value of (0.75-0.65)/(1-0.65) = 0.29. Neurons for which the prediction accuracy was smaller than chance (*a*_*i*_ < *c*_*i*_) therefore had negative proportion explained. We used this proportion explained as the basic measure of accuracy in our data for plotting purposes (Figures 5A–C, 8), though not for finding bias neurons (see below). We also then calculated in which percentile of the chance-level distribution lay the prediction accuracy, and for how many neurons the prediction accuracy lay in the top 50-percentile of their chance-level distribution (Figure 6).

We sought neurons whose pre-cue activity—i.e., activity before the decision alternatives were revealed—was significantly predictive of the animal's choice after cue onset. To accomplish this, we focused on UNDET trials and tested for each neuron whether its pre-cue prediction accuracy was significantly above chance at *p* = 0.05. Therefore, for each neuron we tested whether its UNDET prediction accuracy was among the top 5% of the 10,000 UNDET chance-level values calculated in the bootstrapping procedure above, for the spatial-bias and reward-bias analyses (Figure 6). Neurons that passed this test were designated *bias neurons* and their spiking activity was termed *bias activity*. We tested this bias activity over the pre-cue, cue, and go periods. The go period is defined as the first 500 ms after the onset of the go signal (Figure 1). Similarly, neurons whose UNDET prediction accuracy was among the bottom 5% of chance values (0 of 105, 0%, 5 of 118, 4%, and 1 of 52, 2%, for DLPFC spatial-bias, DLPFC reward-bias, and VS spatial-bias analyses, respectively; all within the proportions expected by chance; Figures 5A–C, 6) were designated *anti-bias neurons*.

#### Error-trials and standardized firing-rate analysis

Trials where both rewards were offered with no delay yet the monkey selected the small immediate reward over the large immediate reward occurred rarely (3% of trials with immediate rewards), and were designated *error trials*. We wanted to understand whether the firing rate of bias neurons for trials in which their preferred direction aligned with the target associated with the smaller reward was higher than over the rest of the trials, which might suggest that the bias activity contributes to committing the error. We therefore searched for such trials recorded for bias neurons and found 24 trials distributed over 7 of the 29 DLPFC spatial-bias neurons, but no trials involving striatal spatial-bias neurons. In order to combine the firing rate across trials from different neurons with different baseline firing rates, we standardized the firing rates by transforming them into *z*-values (i.e., for each neuron separately, we subtracted the mean from each firing rate and divided by the standard deviation).

### Circuit model and simulations

Two neural network circuits were constructed to capture the essential aspects of the neural dynamics of decision-making. The first is a simpler circuit encompassing only spatial bias activity (Figure 9A). The second is full model that includes both spatial and reward-bias activities (Figure S3A), and is described below. The simpler circuit is composed of 5 mean-rate units, modeled as non-saturating half-wave rectified units of the form *f* (*x*) = max (*x* − *T*, 0), where *T* = 1 is a constant activation threshold. Each unit represents the average firing rate of a network of biological spiking neurons (Wong and Wang, 2006). Three of these units (*x*_*L*_, *x*_*R*_, and *h*) form a simple winner-take-all (WTA) network. The other two units bias the activity of the WTA dynamics. The network reaches a decision through competition between excitatory units *x*_*L*_ and *x*_*R*_,—which stand for left and right choice, respectively, and model the population of choice neurons. The choice units are recurrently excitatory (with weight α = 1.5) and mutually inhibitory through a shared inhibitory unit *h* (the connections to/from *h* have the weight β_1_ = 3/β_2_ = 0.4, respectively). This WTA circuit thus suppresses/amplifies the weaker/stronger external input *I*, respectively (Douglas et al., 1995). After convergence only *x*_*L*_ or *x*_*R*_ remain active. The two inputs, *I*_*L*_ and *I*_*R*_, represent the temporally discounted values of the sensory evidence for choosing either the left or right target, respectively. Without any bias activity, the unit receiving the highest discounted value will win (Figure 9B) and determine the direction of the animal's saccade (e.g., through projections of *x*_*L*_ and *x*_*R*_ downstream to motor areas like the frontal eye field or superior colliculus) (Lo and Wang, 2006).

To model the population of spatial bias neurons, we added excitatory bias units, *p*_*L*_ and *p*_*R*_ (implemented as pointer neurons; Hahnloser et al., 1999), which were bi-directionally connected to *x*_*L*_ and *x*_*R*_, respectively (with weight δ = 0.1). The dynamics of the choice units are modeled as τ*ẋ*_*i*_ + *Gx*_*i*_ = *f* (*I*_*i*_ + α*x*_*i*_ + δ*p*_*i*_ − β_1_*h* − *T*_*i*_), and similarly for the other units (see below for detailed equations). Here τ = 0.01 s is the membrane time constant and *G* = 1 is the load. Activation of a bias unit introduces an additional recurrent feedback loop to its choice unit, and thus the effective gain of that choice unit is dynamically increased relative to the other choice unit. Hence, a choice unit with external bias input can win the competition despite having a smaller input value relative to the other choice unit (Figure 9C). The stronger the bias input *b*_*i*_, the more the competition is biased in favor of its corresponding state (Figure 9D). We assume that their activity fluctuates slowly between trials and stems from the positive part of a normal distribution with mean 0 and a small standard deviation. Numerical simulations were performed using Euler integration with Δ = 0.01 s in Matlab (Mathworks, Inc.), with α, β, and δ fixed at the above values. This circuit has a high gain but is nevertheless stable through the balance of excitation and inhibition for a wide range of parameters (Rutishauser et al., 2011).

#### Steady-state analysis of the spatial-bias circuit model

**Figure 7:**
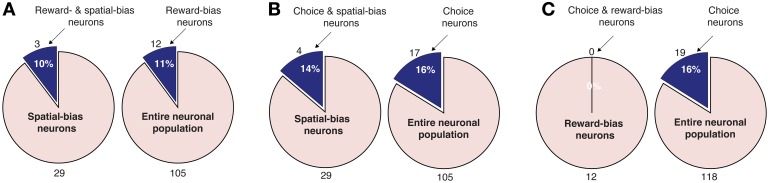
**Pairwise intersections of spatial-bias neurons, reward-bias neurons, and choice neurons in the DLPFC. (A)** The proportion of reward-bias neurons among spatial bias neurons is not significantly different than the proportion of reward-bias neurons among the entire neuronal population (*p* = 0.98, χ^2^-test). **(B)** The proportion of choice neurons among spatial-bias neurons is not significantly different than their proportion among the entire neuronal population (*p* = 0.73, χ^2^-test). **(C)** The proportion of choice neurons among reward-bias neurons is not significantly greater than their proportion among the entire neuronal population (*p* = 0.87, χ^2^-test).

Given the neural network schematized in Figure 7, the dynamic state equations for *i* = *L*, *R* are:

(A.1) $$\tau {\dot{x}}_{i}+Gx_{i}=f\left(I_{i}+\alpha x_{i}+\delta p_{i}-\beta_{1}h-T_{i}\right),$$

(A.2) $$\tau \dot{h}+Gh=f\left(\beta_{2}\sum_{j=L}^{R}x_{j}-T_{h}\right),$$

and

(A.3) $$\tau {\dot{p}}_{i}+Gp_{i}=f\left(\delta x_{i}+b_{i}-T_{p_{i}}\right)$$

Here *b*_*i*_ is the constant bias input into the bias unit *p*_*i*_, which has a two-way connection with its corresponding choice unit *x*_*i*_, for *i* = *L*, *R*. *I*_*i*_ is the external input variable over time into *x*_*i*_ for *i* = *L*, *R*. The firing rate activation function of the units is the non-saturating rectification nonlinearity:

(A.4) f(z)=max(0, z)

If we take *G* = 1 and *T*_*i*_ = *T*_*h*_ = *T*_*pi*_ = *T* for *i* = *L*, *R*, the steady-state value of the winning unit *x*_*w*_ (where *w* is either *L* or *R*) for constant input *I*_*w*_ > 0 and a constant bias *b*_*w*_ > 0 (that is sufficient to activate the bias unit *p*_*w*_) is:

(A.5) $$x_{w}=\frac{I_{w}+\delta \left(b_{w}-T\right)+T\left(\beta_{1}-1\right)}{1-\alpha +\beta_{1}\beta_{2}-\delta^{2}}.$$

Compare this to:

(A.6) $$x_{w}=\frac{I_{w}+T\left(\beta_{1}-1\right)}{1-\alpha +\beta_{1}\beta_{2}}$$

[see also Equation 2.3 in Rutishauser and Douglas (2009)]. Equation A.6. is the steady state activity of the winner choice unit in a network without a bias (i.e., when δ = 0).

Equation A.5 therefore shows that inserting the bias neurons adds two terms to Equation A.6. One is a constant linear term δ(*b* − *T*) multiplied by the gain, 1/(1 − αβ_1_β_2_ − δ^2^), (the gain is the amount by which the choice unit, *x*_*i*_, amplifies its input, *I*_*i*_). The other is a modification (increase) of the gain by the subtraction of δ^2^ from its denominator. Functionally, for the choice unit whose corresponding bias unit is active (that is for *p*_*i*_, *i* = *L*, or *R*, where δ*x*_*i*_ + *b* − *T* > 0), the gain is higher and thus the unit is more likely to win. A choice unit whose bias unit is not active effectively has δ = 0 and is described by Equation A.5, thus ending up with a reduced gain. Hence, for a fixed δ, the only non-linearity is when the bias unit switches from inactive to active; thereafter the network is fully linear in *b* (though non-linear as a function of δ).

#### The full circuit-model

The full circuit model (see Figures S5E,F), encompassing both the spatial and reward-size biases, is an extension of the reduced—spatial-bias-only—circuit-model described in the Materials and Methods (Figure 7). It includes six additional units on top of the five making up the reduced circuit model. Two are the reward-size bias units *q*_*lo*_ and *q*_*hi*_, representing a bias toward the low and high rewards, respectively. These units receive inputs *c*_*lo*_ and *c*_*lo*_, respectively. The other four units are devoted to the conversion of low- and high-reward biases into motion-applicable left or right biases after cue onset. Thus, *q*_*lo*_ is connected *d*_*loL*_ and *d*_*loR*_ and *q*_*hi*_ is connected *d*_*hiR*_ and *d*_*hiL*_, all with connection strength δ_3_ = 5. Then, *d*_*loL*_ and *d*_*hiL*_ are bi-directionally connected to *x*_*L*_ while *d*_*hiR*_ and *d*_*loR*_ are bi-directionally connected to *x*_*R*_, all with connection strength δ_3_ = 0.1. It is important to keep the reward-size bias activity from affecting the choice neurons until the cue signal, which pairs the high and low rewards with left and right targets, reaches the circuit. This pairing is achieved by turning on one of two input signals—either *v*_*hi* = *L*_ or *v*_*hi* = *R*_. Here *v*_*hi* = *L*_ inputs into *d*_*hiL*_ and *d*_*loR*_ while *v*_*hi* = *R*_ inputs into *d*_*loL*_ and *d*_*hiR*_. This is also achieve by keeping the activation threshold of the 4 *d* units a higher than the rest of the units, at *T*_*d*_ = 8. This structure enables the reward-size bias to counter and even later overturn the effects of the spatial bias and the inputs on the choice units (see Figure S5F).

## Results

### Prefrontal and striatal activity related to biases in decision making

The choices of animals that were free to select between a delayed, larger reward and an immediate, smaller reward (Figure 1A) indicated that they increasingly discounted the value of a reward with longer delay (Kim et al., 2008; Hwang et al., 2009) (Figure 1B, Figure S1). Our goal was to study the extent to which the spiking activity of DLPFC, VS, and CD neurons before cue onset predicts the subsequent decision of the animal to select one or the other alternative and how that activity was integrated with the post-cue choice activity. Hence, we tested for correlation and not causation. We hypothesized that such pre-cue bias activity was dissociated from the post-cue choice activity, but that the two would be combined to make a decision at the go signal. Yet, this bias activity would have a weaker influence on the ultimate decision than the choice activity. So the bias activity would mainly have an effect when the values of the decision alternatives were similar.

We therefore divided all trials into four types according to the similarity of the discounted values of the targets, which is to say how much the animal's decision was determined by the stimulus. Trials with most similar target values, and thus relatively undetermined by the reward/delay stimulus, were designated UNDET (see Materials and Methods and Figures 1B, S1). Trials with somewhat similar target values were designated WDET. And trials with more divergent target values were designated SDET. Last, control trials, where the animals were instructed what target to select, were designated *no-choice trials* (NC; see Materials and Methods for details). For neurons coding a bias, the pre-cue activity level (spiking rate) should thus maximally influence decisions between eventual leftward- vs. rightward-movement (or smaller vs. larger reward) in UNDET trials, and that influence should progressively decrease for WDET and then SDET and NC trials.

We investigated two types of bias. The first is a spatial bias, favoring either the target on the left or on the right. The second is a reward-bias, favoring the target with the smaller or larger reward. For each neuron, we calculated the accuracy (proportion of trials with correctly predicted decisions from overall trials) over the four trial types using logistic-regression in a leave-one-out cross-validation scheme (see Materials and Methods; Figures 2–4 show raster plots, spike-density functions and the best-fit logistic regression for DLPFC spatial bias, DLPFC reward bias, and VS spatial bias, respectively). As we were interested in bias activity, we focused on the 1.5 s before cue onset. Using bootstrapping, we then computed the chance level for each neuron, from which we could compute the proportion of the variance that the neuron's activity explained above chance level for every trial type (hereafter, *proportion explained*; see Materials and Methods). We describe the results from 105 DLPFC neurons, 52 VS neurons, and 54 CD neurons for spatial-bias calculations. For the reward-bias analysis we included 13, 4, and 6 additional DLPFC, VS, and CD neurons that met the analysis criteria, respectively (see Materials and Methods).

We expected the pre-cue firing rate of bias neurons (henceforth *bias activity*) to be most predictive of the decision during UNDET trials. The accuracy in UNDET trials for spatial bias was significantly greater than chance for 29 DLPFC neurons (28%) and 14 VS neurons (27%), while 12 DLPFC neurons (10%) showed significant reward bias (at *p* = 0.05, see Materials and Methods; Figures 5, 6, Table S1). These were designated *DLPFC spatial-bias neurons*, *VS spatial-bias neurons*, and *DLPFC reward-bias neurons*, respectively. These numbers of bias neurons are significantly larger than expected by chance (*p* = 3 · 10^−14^, *p* = 2 · 10^−7^, and *p* = 0.015, binomial test, respectively; Figures 5A–C). As a control, we ran the same bias-neurons search procedure for SDET trials, where the stimulus should be the overwhelming influence on the decision. We therefore did not expect to be able to extract a significant number of bias neurons there. Indeed, we found only 8 DLPFC spatial-bias neurons and 3 VS spatial-bias neurons, which is within what would be expected by chance (*p* = 0.16 and *p* = 0.49, binomial test, respectively).

To make sure that our results are not due to our selection criteria for bias neurons, we also examined the entire neuronal populations for spatial and reward biases in the DLPFC and for spatial bias in the VS. We calculated the percentile of the prediction accuracy of each neuron in the population in its chance-level distribution (see Materials and Methods). And we found that the mean of the percentiles for the 105 neurons that took part in DLPFC spatial-bias calculations, the 118 neurons that participated in DLPFC reward-bias calculations, and the 52 neurons that were involved in VS spatial-bias calculations was 66, 57, and 63%, respectively; all significantly greater than the corresponding chance-distribution means (*p* = 2 · 10^−7^, *p* = 0.01, and *p* = 0.004, respectively; Wilcoxon tests; Figure 6). The numbers of neurons in each population in the top 50-percentile—69 of 105 for DLPFC spatial-bias analysis, 70 of 118 for DLPFC reward-bias analysis, and 33 of 52 for VS spatial-bias analysis—were also significantly above chance for all three populations (*p* = 8 · 10^−4^, *p* = 0.026, and *p* = 0.035, respectively; binomial tests; Figure 6).

In addition, we expected a progressive decrease in the predictive power of the bias activity as the difference in the temporally discounted values of the two targets increased. We therefore calculated the predictive power of the bias neurons also over WDET, SDET, and NC trials. And we found the expected decrease in our data from UNDET trials, through WDET, to SDET and NC trials (Figures 5D–F). We conclude that the neural activity of the 29 DLPFC and 14 VS spatial-bias neurons as well as 12 DLPFC reward-bias neurons predicted the upcoming choice of movement direction or reward size before any information about the reward-delay combination of the alternatives became available to the animal.

**Figure 8:**
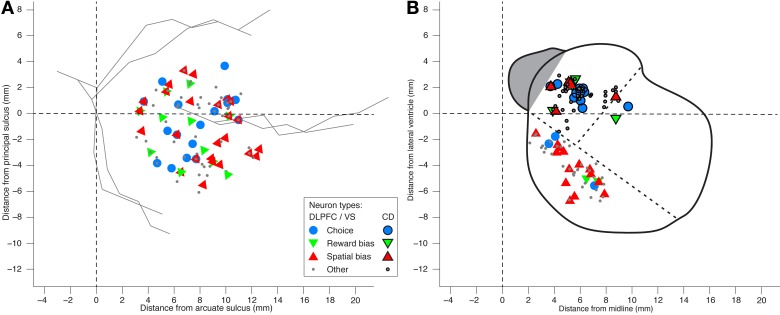
**Locations of neurons recorded in DLPFC and striatum. (A)** Anatomical locations of the DLPC neurons. Distance from the cortical surface is not shown in this planar plot. Hence neurons that differ only in their depth appear on top of each other, as do neurons that belong to more than one group. No evidence was found for anatomical clustering of the various neural groups (1-way MANOVA *p* > 0.15 for all groups). **(B)** A coronal view of neuron locations in ventral striatum (VS; black marker edges) and caudate nucleus (CD; no marker edges). Dotted lines designate the border between CD, putamen, and VS.

We also analyzed the data in search of choice neurons. Using a regression model that included the temporally discounted values of both targets, we discovered that 17 of the 105 DLPFC neurons (16%; *p* = 2 · 10^−5^, binomial test) and 12 of the 54 CD neurons (22%; *p* = 1 · 10^−5^, binomial test) had significantly altered activity during the cue period (Figure 1A) according to the animal's choice of target (Table S1) (Kim et al., 2008; Cai et al., 2011). We refer to them as *choice neurons*. It was of interest to investigate to what extent spatial-bias neurons were also reward-bias neurons and choice neurons, and vice versa. In the DLPFC, the intersection of spatial- and reward-bias neurons with the choice neurons was not significantly larger than would be expected by chance when these effects were combined independently (*p* = 0.73 and *p* = 0.87, respectively, χ^2^-test; Figures 7B,C), indicating that choice neurons were not preferentially also bias neurons of either kind. Moreover, we found no evidence for anatomical clustering of spatial or reward-bias neurons or of choice neurons in the DLPFC of the two animals (Figure 8A). Unlike the DLPFC, in the striatum we found that only VS contained a significant number of spatial-bias neurons, and the proportion of spatial bias neurons was significantly higher in VS than in CD (Figure 8B, Table S1). In addition, only the CD contained a significant number of choice neurons, and the proportion of such neurons was again significantly greater than that of these neurons in VS. Neither VS nor CD contained a significant number of reward-bias neurons (Figure 8B, Table S1).

#### False-negatives analysis

We used logistic-regression in a leave-one-out cross-validation procedure to keep the false-positives rate for discovering bias neurons low. This comes at the expense of an increased probability of mistakenly rejecting neurons that are actually bias neurons, thus increasing the false-negative rate. We therefore developed a method to estimate a lower bound on this false-negatives rate. Given our recording procedure, we generally have acces to only a single neuron at a time, and always to only one bias neuron at a time. And in our limited sample of trials, activity patterns of a true bias neuron might, due to noise for example, not coincide with that of the overall bias population that—according to our model—determines the decision of the monkey. To assess this false-negatives rate, we calculated the preferred and non-preferred reward size and the number of trials associated with each DLPFC reward-bias neuron. Taking the mean firing rate and number of trials for the preferred and non-preferred reward sizes, we ran a simulation, sampling from a Poisson process with these firing rates and number of trials over a 1.5 s duration (the duration of our pre-cue period). We ran our bias-neurons identification procedure on the simulated results to test whether such a simulated reward-bias neuron would be identified by our procedure. We ran this bootstrapping procedure 1000 times, and for the DLPFC reward-bias neurons found that in only 70% of these runs the simulated neuron was identified as a bias neuron. Therefore, our population of 118 neurons involved in reward-bias analysis may in fact contain 12/0.7 ≈ 17 reward-bias neurons instead of only 12 bias neurons.

Importantly, other factors that are harder to simulate may increase the false-negatives rate: The recorded neuron might belong to, say, the high-reward bias population and we may happen to record a majority of trials where the monkey opted for the low reward in our limited sample. Alternatively, interactions between reward-bias neurons and spatial-bias neurons may cancel each other out, especially in a limited sample (Figure S3B). Therefore, the 70% of trials where a simulated reward-bias neuron was correctly identified should be viewed as an upper bound on the false-negatives rate inherent in our procedure, and it is possible that there are more than 17 reward-bias neurons in the recorded neuronal population.

### Potential sources of the bias activity

Our analysis suggests that a significant number of DLPFC and VS neurons show single-trial pre-cue biases. We tested and excluded several possible sources of this bias activity. If the animal's current choice, especially for UNDET trials, is significantly correlated with its choice in the preceding trials, the pre-cue biases might be a reflection of persistent activity associated with the choice in the previous trial or of behavioral choice patterns across trials. But we found no evidence for correlations between choices in UNDET and preceding trials, nor between choices when examining only UNDET trials (as far as 10 trials back; Supplemental Text, Figure S4). In addition, we often found left/right and small-/large-reward differences in the bias activity throughout the 1.5 s pre-cue period (see examples in Figures 2–4). Had the bias activity reflected lingering neuronal activity from the previous trial, we would have expected to find these differences mainly in the beginning of the pre-cue period. This also suggests that it is not only bias activity close to cue onset that affects the animal's eventual decision.

For each neuron we also calculated whether its firing rate tended to be higher for all trials culminating in leftward (or rightward) saccades for spatial-bias neurons or in saccades toward the smaller (or larger) reward for reward-bias analysis, during the pre-cue epoch. We found that 17 of the 29 DLPFC spatial-bias neurons and 9 of the 14 VS spatial-bias neurons were more active toward the left, and that 7 of the 12 reward-bias neurons were more active toward the small reward; none of these are significantly different from an even split (*p* = 0.23, *p* = 0.21, and *p* = 0.39, binomial test, respectively). We therefore found no evidence that bias neurons had a preferential preferred direction.

Lastly, in the DLPFC, 8 (9) of the 11 (18) bias neurons that were recorded from the left (right) hemisphere—i.e., recorded from animal D (J) (see Figure 6A)—had higher firing rate toward the left (right), across all trials in the pre-cue epoch. There was therefore no significant correlation between the hemispheric laterality and the target toward which the neuron was more active (*p* = 0.34 and *p* = 0.29, χ^2^-test of 8/11 vs. 17/29 and of 9/18 vs. 11/29, respectively). So we could not find evidence for correlation between the brain hemisphere from which the DLPFC neurons were recorded and their preferred direction. All VS neurons were recorded from the right hemisphere (Figure 6B). Following this and the even split for left/right preferred directions reported above, our data shows no spatial preference in terms of the recorded brain hemisphere and the behaviorally chosen side.

### Predictions from a circuit model

**Figure 9:**
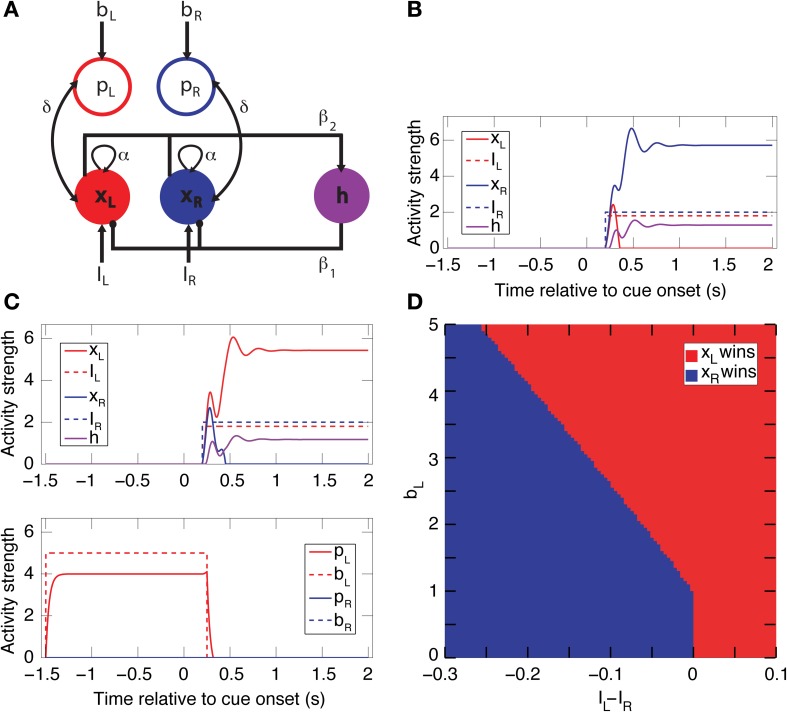
**A simple biased WTA circuit model accounts for the spatial-bias data and makes additional predictions. (A)** A circuit for spatial-bias activity. Units *x*_*L*_ and *x*_*R*_ designate populations of left and right choice neurons, which are self-excitatory and mutually inhibitory (through inhibitory neuron *h*). Each choice unit is bi-directionally connected to a bias unit (*p*_*L*_ and *p*_*R*_) Excitatory (inhibitory) connections are designated by arrows (circles). **(B)** Without bias input (*b*_*L*_ = *b*_*R*_ = 0), this is a simple WTA network, so for inputs *I*_*R*_ > *I*_*L*_ (*I*_*R*_ = 2 and *I*_*L*_ = 1.8, in this case) *x*_*L*_'s activity diminishes to zero and *x*_*R*_'s increases and then stabilizes. **(C)** When the left bias is turned on (*b*_*L*_ = 5, *b*_*R*_ = 0), *x*_*L*_ now wins for the inputs in B. Note that the bias activity was turned off (at *t* = 250 ms) shortly after the onset of the competition (at *t* = 200 ms). **(D)** A phase-space diagram simulating various values for *b*_*L*_ and *I*_*L*_ − *I*_*R*_ when *b*_*R*_ = 0 and *I*_*R*_ = 2. The separatrix between the basin of attraction of *x*_*L*_ winning and *x*_*R*_ winning is constant until approximately 1, when *b*_*L*_ passes its activation threshold, and then linear. (See Figure S3 for the full model, including both spatial- and reward-bias activity.).

To better understand the nature of this pre-cue bias activity, we developed a plausible neural circuit model of a biased WTA network (Figure 9A, Materials and Methods). Unlike a simple WTA circuit where the identity of the winner depends only on the inputs (Figure 9B), in our model the activity of a divergent population of bias units also influences the winner selection (Figures 9C,D). Hereafter we refer to the elements of the circuit model as *units* and to actual DLPFC or BG cells as *neurons*; each unit represents a population of neurons. The model had two versions—one that focused on the spatial bias activity alone (Figure 9) and another, expanded version encompassing both spatial and reward biases (Figures S3A,B). The model does not incorporate noise for the sake of simplicity. Nevertheless, simulations we performed indicate that, even in the presence of noise, the results remain the same on average.

**Figure 10:**
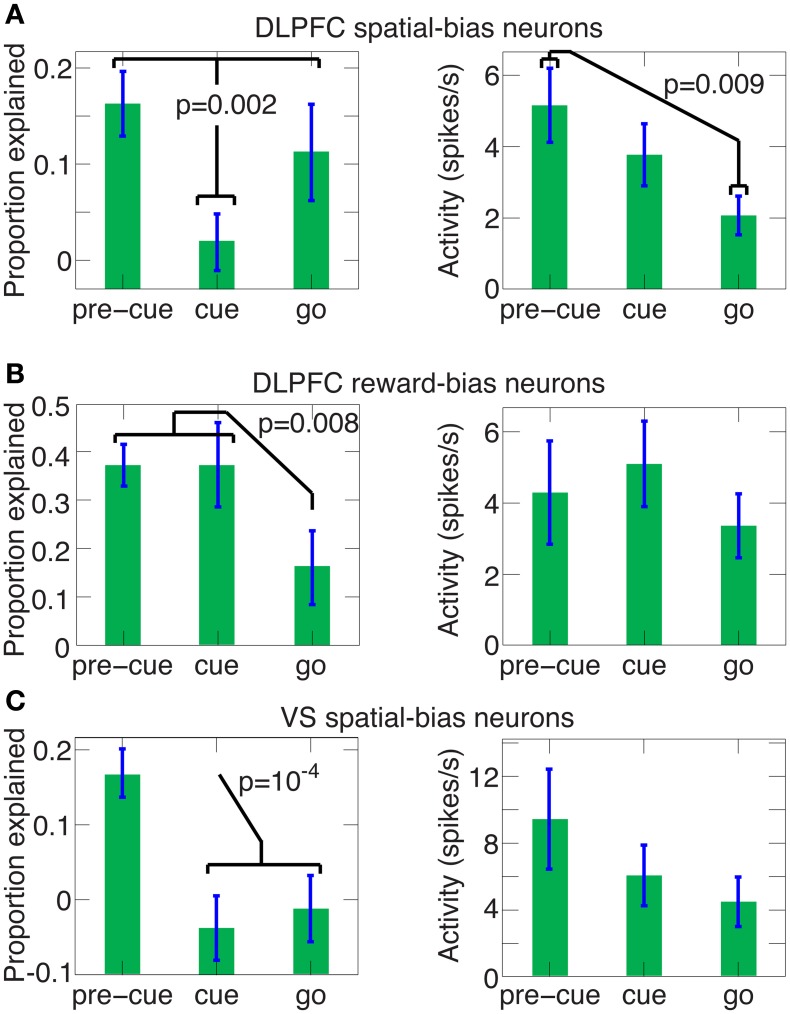
**The predictive power of spatial and reward DLPFC bias neurons and VS spatial-bias neurons during the pre-cue, cue and go periods.** The average (±SEM) proportion explained above chance level and the average (±SEM) activity levels are depicted for the pre-cue, cue and go period for all 29 DLPFC spatial-bias neurons (in the left and right panels of **A**, respectively), for all 12 DLPFC reward-bias neurons (in the left and right panels of **B**, respectively), and for all 14 VS spatial-bias neurons (in the left and right panels of **C**, respectively). *P*-values in the figure are for one-tailed *t*-tests. **(A)** The proportion of accuracy explained in the pre-cue and go periods are not significantly different (one-tailed *t*-test *p* = 0.2), but are significantly larger than in the cue period, where the proportion drops to chance-level (one-tailed paired *t*-test *p* = 0.25). In contrast, the activity of the neurons in the go period is significantly weaker than in the pre-cue period. **(B)** The proportion explained for the pre-cue and cue periods are not significantly different (one-tailed *t*-test *p* = 0.5), but a significant decrease occurs for the go period, which is at chance level (one-tailed *t*-test *p* = 0.2). There is no significant trend in the activity levels among the pre-cue, cue, and go periods (one-way ANOVA *p* = 0.64). Note that the proportion explained for the DLPFC reward-bias pre-cue period is also significantly higher than the pre-cue proportion explained in DLPFC spatial bias (in **A**, one-tailed *t*-test *p* = 6 · 10^−4^). **(C)** The proportion explained drops significantly after the pre-cue period to chance level (one-tailed paired *t*-test *p* = 0.8 and *p* = 0.6 for the cue and go periods, respectively). Again there is no significant trend in the activity levels across the pre-cue, cue, and go periods (one-way ANOVA *p* = 0.3).

A main thrust of our model is that the bias and choice units are dissociated, as indicated by our data: We discovered that bias neurons are not preferentially also choice neurons in DLPFC (Figures 7B,C, 8A). And we found that, of the three types of neurons, there are only spatial-bias neurons in significant numbers in VS and only choice neurons in significant numbers in CD (Figure 8B). Our simulations also demonstrate that it is sufficient for the spatial-bias units to be active at the very beginning of the competition to influence the identity of the final winner: After giving an initial boost to the network dynamics, the WTA network amplifies any initial small differences. We therefore stop the input to the model's bias units shortly after the onset of the inputs to the choice units (Figure 9C). Our circuit model thus suggests that the prediction accuracy of the spatial-bias neurons may decrease with time during the cue period. This prediction is of interest because it relates to the manner by which the bias and choice activities are combined after cue onset by postulating how bias neurons would behave after cue onset, when rational deliberation can begin. We tested this model prediction by further analysis of our experimental data during the cue period and the first second after the go signal, and found it to be true. For DLPFC spatial-bias neurons, there was no significant decrease in the proportion explained between the pre-cue period and go period, while the proportion explained during the cue-period decreased to chance level (Figure 10A). For VS spatial-bias neurons, the proportion explained decreased to chance level during both the cue and go periods (Figure 10C). We think that this might be because DLPFC spatial-bias neurons may be more involved in the motor aspects of the task than VS spatial-bias neurons.

For reward biases, in contrast, the mapping between the high and low rewards and the left and right targets is only revealed by the stimulus. So, reward-biases can be translated into spatial coordinates only after stimulus onset (see full model, Figure S3A). To influence the decision effectively, the reward-bias thus needs to remain active longer than the spatial bias (Figure S3B). The neural data we gathered also reflects this; the DLPFC reward-bias neurons are the only ones remaining predictive during the cue-period (compare Figure 10B and Figures 10A,C).

#### “Error-trials” analysis

In value-based decisions, it is generally not possible to know with certainty whether the chosen target was associated with the subject's larger discounted value or not. Except in some special circumstances (see below), it is thus not possible to declare a choice correct or incorrect. However, when both the small and large rewards are offered with no delay (an SDET trial), animals maximizing the temporally discounted value of reward (Figure 1B) should always select the larger immediate reward (which they did for 97% of 1536 and 99% of 1698 of such DLPFC and striatal trials, respectively). Following the model prediction above, immediate reward trials where the animal nevertheless chose the small reward—hereafter *error trials*—should be associated with especially strong bias activity, and hence with higher firing rate of the bias neurons. Consistent with this prediction, the mean (±SEM) standardized firing rate (see Materials and Methods) over all the DLPFC spatial-bias neurons whose preferred direction aligned with the target associated with the smaller discounted value during error trials was 0.68 ± 0.44, which was significantly higher than their standardized firing rate during the rest of the trials, at 0 ± 0.03 (one-tailed *t*-test *p* = 0.008; the value of 0 for the mean firing rate results from the standardization procedure, see Materials and Methods). The error trials above were distributed throughout the experimental sessions, suggesting that the animal's motivation level and alertness did not factor into the firing rate difference. The striatum data contained too few error trials for similar statistical analysis.

## Discussion

We examined single-neuron activity from the DLPFC, VS, and CD of monkeys that freely choose between a delayed, larger reward and a more-immediate, smaller one. Our working hypothesis was that such activity before the cue revealed the decision alternatives—and thus before rational deliberation could begin—had some influence on the ultimate choice of the animals. We speculated that such bias activity was dissociated from the post-cue choice activity, and had a weaker influence on the decision than that choice activity. So, when it was later integrated with the choice activity, the effect of the bias activity would be mainly apparent where the reward/delay combinations would have similar values for the animal (i.e., in UNDET trials, in our case).

We found that when the animals were confronted with two targets with similar temporally discounted values for the rewards associated with the targets, more than a quarter of the analyzed neurons in DLPFC and in VS signaled the direction of the eventual eye movement before cue onset. In addition, 1 in 10 DLPFC neurons coded the eventual choice of the animal between a larger or smaller reward—or equivalently, between the red and green targets, respectively—before cue onset. In such trials, a choice between the large and small reward usually also translated into a choice between the short and long delay, respectively. Moreover, we found significant numbers of both types of bias neurons as well as of choice neurons in the DLPFC; both types of bias neurons were not preferentially also choice neurons, nor were spatial-bias neurons preferentially also reward-bias neurons, and vice versa. Yet, we found only spatial-bias neurons in significant numbers in the VS and only choice neurons in significant numbers in the CD. The spatial- and reward-bias activities were found, on average, over the entire DLPFC neuronal population, as was the spatial-bias activity found on average over the entire VS population. This suggests that the presence of bias neurons was not merely an artifact of our selection criteria. Our results are therefore compatible with our hypothesis.

We considered the implications of the presence of bias neurons for decision-making using a computational approach. In our task, we viewed decision making as a competition between the two choices represented by the targets. And this choice activity exerted a stronger influence on the results than the bias activity. The function of the bias neurons was to more-weakly favor one of the alternatives, thus making that choice more likely, especially when the choice activity only marginally swayed the competition. While there are different approaches and levels of abstraction to modeling such decision-making, almost all regard making a decision as a competition. Drift-diffusion and race-to-threshold models (Smith and Ratcliff, 2004) have been used extensively and account for data acquired in perceptual discrimination tasks (Shadlen and Newsome, 2001; Mazurek et al., 2003; Palmer et al., 2005; Heekeren et al., 2008) as well as value-based decisions (Basten et al., 2010; Krajbich et al., 2010). However, their abstract mathematical nature does not easily lend itself to exploration of circuit mechanisms. In particular, it is not clear how to represent choice and bias neurons as dissociated populations (Figure 9A, Figure S3A) in these more abstract models. In contrast, biophysically based cortical microcircuit models for decision-making based on attractor dynamics explain decision-making data well (Wang, 2002; Wong and Wang, 2006), while suggesting mechanisms for the instantiation of these decisions in neural substrate (Rolls and Deco, 2011). We thus focused our modeling efforts on circuits implementing competition using shared inhibition between two pools of competing excitatory units. In this framework, bias neurons can be included naturally as additional units that bias the competition between different pools.

We derived several empirically testable predictions from our model, which we in turn tested in the data. First, the model suggested that spatial-bias, reward-bias, and choice neurons might be pairwise-divergent populations. We found that this was the case in the striatum, with the VS and CD containing only spatial-bias and choice neurons in significant numbers, respectively (Figure 8B). In the DLPFC we found that choice neurons are not preferentially also either type of bias neurons, nor are spatial-bias neurons preferentially reward-bias neurons, and vice versa (Figure 7). Moreover, we found different activity patterns for DLPFC spatial-bias, reward-bias and choice neurons across the pre-cue and cue periods (Figure 10). In that vain, the model also suggested that while spatial-biasing activity is required only in the beginning of the competition, the reward-biases would remain on longer after cue onset (Figure S3B). Also inline with our model's predictions, in “error” trials (where the animals chose a small immediate reward over a large immediate one) the firing rate of the bias neurons, whose preferred direction aligned with the target associated with the smaller temporally discounted choice, was higher than in other trials. However, as only a small number of neurons and trials were involved in this analysis, this result can only be taken as preliminary evidence that bias activity may occasionally also affect the monkey's decision when the values of the two decision options are substantially different.

In other tasks, when the most rewarding choice could be calculated before cue onset, predictive pre-cue neural activity was found in CD choice neurons (Coe et al., 2002; Lauwereyns et al., 2002; Ding and Hikosaka, 2006). In contrast, in our task where the most rewarding choice could only be deliberated upon after cue onset (due to the random shuffling of the reward size and delay among trials), we found only post-cue CD choice activity. Combined, these data are therefore consistent with the hypothesis that deliberation about an upcoming choice, be it before or after cue onset, involves choice neurons (in DLPFC and CD, in our case), while pre-deliberation bias activity involves bias neurons (spatial ones in DLPFC and VS and reward ones in DLPFC alone).

Other studies utilized a perceptual judgment task (random-dot motion discrimination) to study choice activity. In that task, pre-deliberation spatial-bias activity was found in monkey LIP (Shadlen and Newsome, 2001; Williams et al., 2003) and CD (Ding and Gold, 2010), but not in DLPFC (Kim and Shadlen, 1999). There are three important differences between those findings and ours. First, while in perceptual judgments the reward is usually contingent upon the correct response to the perceptual cue, for value-based decisions there are seldom correct and incorrect answers and the animal is free to choose its reward. Second, previous studies reported spatial biases whereas we also found non-spatial biases that reflect preferences for reward size and are dissociated from spatial bias activity. Third, these studies focused on pre-cue bias activity in choice neurons, while we searched for bias activity in the entire neural population, and found bias activity that was dissociated from choice activity in DLPFC, and with anatomical specificity for bias and choice neurons in the striatum. We need not commit to a strict distinction between perceptual and value-based decisions, but the above may explain why we found DLPFC bias activity when previous studies did not and why we found only choice activity in CD and others found pre-cue spatial-bias activity in a small fraction of choice neurons there (Ding and Gold, 2010). Also, whereas a fluctuating bias may reduce the reward in a perceptual decision task, it may well be part of the subjective aspect of value-based decisions (Gold and Shadlen, 2007). Last, our animals might have used deliberative goal-directed judgments to accomplish their task, while subjects making perceptual decisions relied on intuitive-habitual judgments, which may involve different brain systems (Daw et al., 2005; Rangel et al., 2008).

The bias activity we recorded does not seem to be related to past behavioral patterns, nor does it have preferential preferred direction or hemispheric specificity. This is in contrast to neural activity in forced-choice memory tasks, where striatal neurons, but not DLPFC neurons, exhibit strong contralateral spatial preference (Kawagoe et al., 1998; Kobayashi et al., 2007). Moreover, the structure of our task, together with the random shuffling of the reward size and delay across trials, left no beneficial role for anticipation (Lauwereyns, 2010). Nor is it likely that visual attention played a role in bias formation, because during the pre-cue period the screen was empty but for a small central fixation target. But, as we found that DLPFC spatial-bias neurons were similarly predictive during the pre-cue and go period, yet not predictive during the cue period (Figure 10A), this DLPFC bias activity may include a motor component. Going back to the question with which we started, it seems that in our experimental setting, the bias activity is unlikely to be decision related, as it is recorded before the decision alternatives are presented and rational deliberation can begin. It may be related to top-down attention (Hopfinger et al., 2000; Changizi and Shimojo, 2008), or to other factors not immediately related to the neural mechanisms of decision making. In particular, the single-trial pre-cue bias activity we found may be related to slowly fluctuating spontaneous brain activity (Fox et al., 2007) thought to be involved in responding to stimuli in conjunction with stimulus-evoked activity (Arieli et al., 1996; Wang, 2002; Leopold et al., 2003; Ress and Heeger, 2003; Fox et al., 2006; Vincent et al., 2006). If the bias activity is dependent on such fluctuations, it could explain why the bias and choice neurons may be distinct populations: the bias neurons may belong to a network of spontaneous activity, distinct from the decision network. A recent model of the neural substrates underlying unmotivated free-choices suggests that the onset of such choices depends on spontaneous fluctuations in neuronal activity (Schurger et al., 2012). So, assuming that the bias-neuronal activity we found is based on spontaneous fluctuations, might suggest that another aspect of free actions—action selection—also relies on these fluctuations. Neural fluctuations as the basis of our results are also congruent with the finding that inhibiting DLPFC activity in humans using transcranial magnetic stimulation reduces their ability to generate random motor sequences (Jahanshahi and Dirnberger, 1999). Moreover, it is inline with the proposed role of the BG and frontal cortex in biasing competitions among neural activations, which originate from sensory information and result in action (Cisek and Kalaska, 2010). Perhaps while the VS holds predeliberation biases, the CD informs the decisions, as they are made, through choice neurons.

As for testing the predeliberation-biases hypothesis, do decisions—especially indeliberate ones, between similarly valued options—also rely on predeliberation activity to bias the choice toward one of the alternatives? Our results appear consistent with that hypothesis. And, as the model demonstrates, even weak bias activity can end up greatly influencing the identity of the selected decision alternative. What is more, our results suggest that these bias brain-signals may not be part of a decision process that is normally involved in rational deliberation, because they happen before the decision alternatives are revealed and rational deliberation can begin. Instead, the bias signals we measured may depend on a dissociated predeliberation network. Raising the left or right hand for no reason or purpose and with little consequence (Haggard and Eimer, 1999; Haynes et al., 2007; Soon et al., 2008, 2013; Bode et al., 2011; Fried et al., 2011) is the epitome of such random, indeliberate decisions. So the weak-to-moderate prediction ability well before movement onset that is exhibited in these experiments (60–70% accuracy) might rely on such fluctuating bias activity rather than be part of the decision network normally involved in rational deliberation. It is therefore of interest that some recent attempts to model indeliberate decisions focus on the DLPFC as the locus of intentional action selection (Pockett, 2006; Pacherie and Haggard, 2011). Further research is required to shed more light on the origins of this early prediction ability.

But similarly valued decision options are not restricted only to such random choices. Arguably, some of the biggest and toughest decisions in life—such as selecting a partner or career path—are difficult also because the decision alternatives are associated with similar values (e.g., is a job that is better paying but less interesting and more demanding than the current one preferable?). Our results—to the extent they can be generalized to such situations—suggest that these decisions may well end up being considerably influenced by neural biases that are not part of the rational decision process and start before rational deliberation can even begin.

### Conflict of interest statement

The authors declare that the research was conducted in the absence of any commercial or financial relationships that could be construed as a potential conflict of interest.
