# Supplementary material for: Predeliberation activity in prefrontal cortex and striatum and the prediction of subsequent value judgment
Source: Front Neurosci. 2013 Nov 26;7:225. doi: 10.3389/fnins.2013.00225 (PMC3840801; doi:10.3389/fnins.2013.00225)
Supplement: Supplementary file 1 [file Presentation1.PDF]

Pre-deliberation activity in prefrontal cortex and striatum and the prediction of subsequent value judgment

Uri Maoz, Ueli Rutishauser, Soyoun Kim, Xinying Cai, Daeyeol Lee and Christof Koch

Supplemental Information

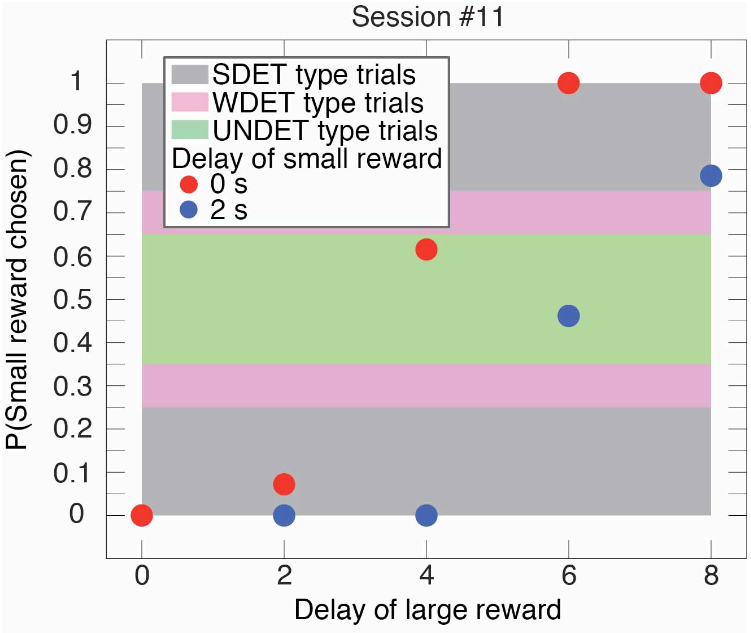

Figure S1: The animal's choice behavior for one sample session (related to Fig. 1). A representative session from the overall behavior depicted in Figure 1B. Choice trials are divided into three types – UNDET, WDET and SDET – according to the empirical probability of selecting the smaller reward from a given pair of small and large rewards and their associated delays.

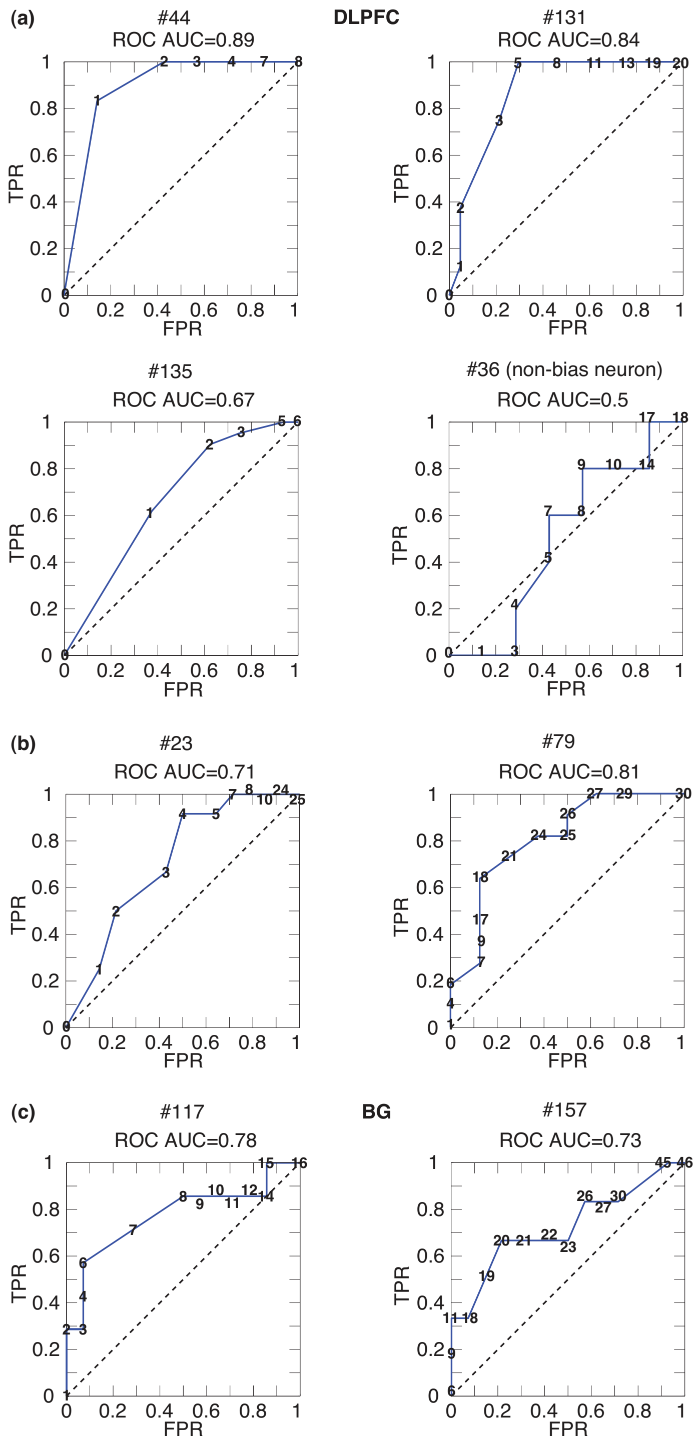

Figure S2: Examples of ROC plots for various types of neurons (related to Figs. 2-4). The receiver-operating characteristic (ROC) curves of the DLPFC spatial-bias neurons from Figure 2 in A, DLPFC reward-size bias-neurons from Figure 3 in B and VS spatial-bias neurons from Figure 4 in C. TPR (FPR) is the true-positive rate (false positives rate) – i.e., proportion of true positives (false negatives) from all positive (negative). The area under the curve (AUC, or choice probability) for each panel is given above it. Higher choice-probability values generally correspond to higher prediction accuracy computed using logistic regression.

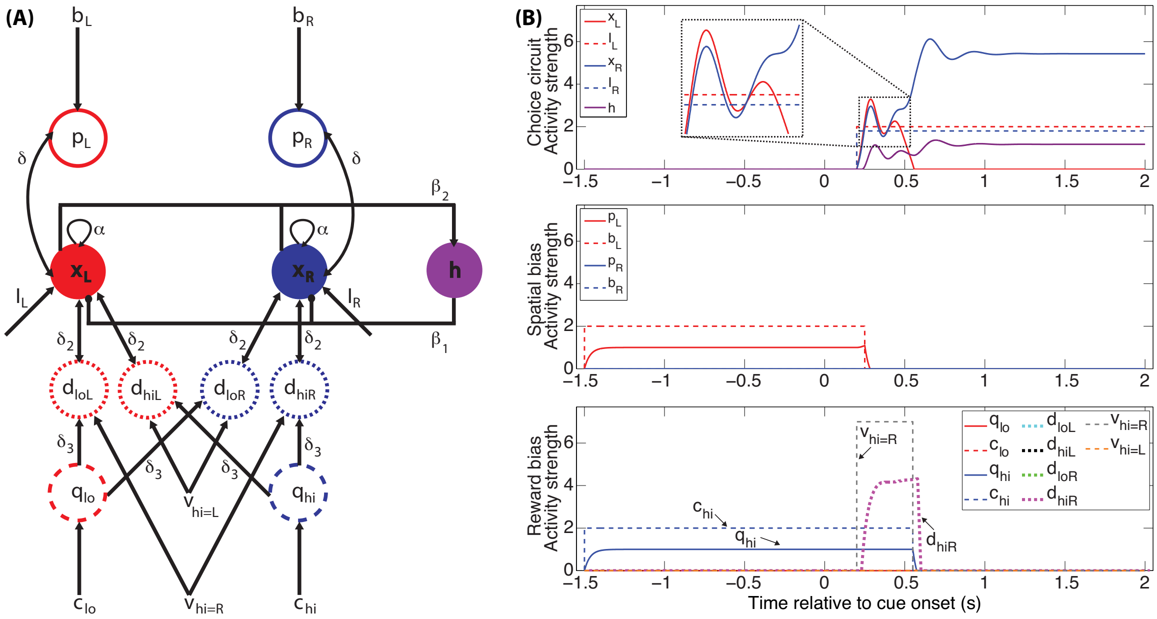

Figure S3: Simulations of the full model (related to Fig. 7). (A) A diagram of the full model, which includes the partial spatial-bias model depicted in Figure 7A on the top with the circuitry to add the reward-size bias functionality on the bottom. Here *L*, *R*, *hi* and *lo* stand for left, right, high-reward and low-reward, respectively. (B) The spatial- and reward-biases both affect the data before cue onset. A simulation of the model with the winner-take-all circuitry that includes the choice units in the top panel, the spatial-bias activity in the middle, and the reward-size bias activity in the bottom. Notice that $I_L > I_R$ and $b_L=2$ while $b_R=0$. Hence, in the beginning of the competition, x_L rapidly grows larger than x_R because of the spatial bias – see inset on top panel. But the reward bias, $c_{hi}=2$ while $c_{lo}=0$, and this – together with $V_{hi=R}$ turning on at the same time as I_L and I_R (at t=0.2s) – eventually makes $x_R > x_L$, and thus x_R ultimately wins the competition.

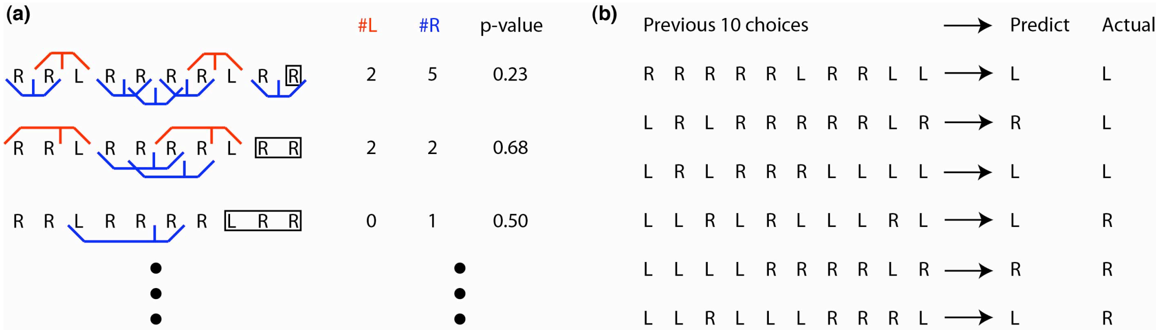

Figure S4: Some examples of the behavioral prediction algorithm. The examples here refer to L/R (left/right) choices. But the algorithm works similarly for high-/low-reward choices. (A) An example of a sequence of choices from our data. Attempting to find the next choice based on behavioral history, the algorithm counts how many times the last choice (R in this case) was followed by R (5 in this case, in blue) versus L (twice, in red) and finds the p-value of the binomial test for this distribution for L and R trials when assuming uniformity (i.e., assuming p(L)=p(R)=0.5). It then counts how many times the last two choices (RR in this case) were followed by R (twice) versus L (also twice) and calculates the p-value, and so on for longer patterns until no more instances of the pattern can be found in the history. It then finds the minimal p-value and uses it to generate its prediction. The algorithm looks through the history of the last 10 choices. (B) Six examples of the predictions of the algorithm for sequences of left/right choices from our data and the actual next choice.

	DLPFC	VS	CD
Spatial-bias neurons	**29/105=0.28** ($p=3\cdot10^{-14}$)	**14/52=0.27** ($p=2\cdot10^{-7}$)	5/54=0.09 (p=0.13)
Reward-bias neurons	**12/118=0.10** (p=0.015)	2/56=0.04 (p=0.78)	4/60=0.07 (p=0.35)
Choice neurons	**17/105=0.16** ($p=2\cdot10^{-5}$)	3/52=0.06 (p=0.45)	**12/54=0.22** ($p=1\cdot10^{-5}$)

Table S1: Numbers and proportions of the various types of cells in the different brain areas. The number and proportion of spatial-bias neurons, reward-size bias neurons and choice neurons in the DLPFC, VS and CD. In bold are entries where the number of bias or choice neurons out of the overall number of neurons in that area is more than expected by chance ($p<0.05$, binomial test). The proportions of spatial-bias neurons in DLPFC and VS are not significantly different (p=0.87), but are both significantly greater than the proportion of spatial bias-neurons in CD ($p=8\cdot10^{-11}$ and $p=1\cdot10^{-5}$, respectively). Similarly, the proportion of choice neurons in DLPFC and CD, while not significantly different (p=0.14) are both significantly greater than in VS ($p=5\cdot10^{-6}$ and $p=2\cdot10^{-7}$, respectively). The proportion of reward-size bias-neurons in DLPFC is significantly greater than in VS ($p=1\cdot10^{-4}$; significance tests carried out with χ^2 test).
